# MEDUCATE trial: effectiveness of an intensive EDUCATional intervention for IT-mediated MEDication management in the outpatient clinic – study protocol for a cluster randomized controlled trial

**DOI:** 10.1186/s13063-015-0744-8

**Published:** 2015-05-22

**Authors:** F. van Stiphout, J.E.F Zwart-van Rijkom, J.E.C.M. Aarts, H. Koffijberg, E. Klarenbeek-deJonge, M. Krulder, K.C.B. Roes, A.C.G. Egberts, E.W.M.T. ter Braak

**Affiliations:** Department of Internal Medicine and Centre for Research and Development of Education, University Medical Centre Utrecht, the Netherlands, Hijmans van den Berghgebouw kamer 4.21, Huispostnummer HB 4.05, Postbus 85500, 3508 GA Utrecht, The Netherlands; Department of Clinical Pharmacy, University Medical Centre Utrecht, Heidelberglaan 100, 3584 CX Utrecht, The Netherlands; Division of Pharmacoepidemiology and Clinical Pharmacology, Utrecht Institute for Pharmaceutical Sciences, Faculty of Science, Utrecht University, PO Box 80082, 3508 TB Utrecht, The Netherlands; Institute of Health Policy and Management, Erasmus University Rotterdam, Rotterdam, The Netherlands; Department of Biomedical Informatics, School of Medicine and Biomedical Sciences, University at Buffalo, Roosevelt Hall, 923 Main Street, Buffalo, NY 14203 USA; Department of Health Technology Assessment, Julius Center, University Medical Centre Utrecht, Utrecht, The Netherlands; Faculty of Medicine, Utrecht University, Universiteitsweg 98, 3584 CG Utrecht, The Netherlands; Department of Quality and Patient Safety, University Medical Centre Utrecht, Utrecht, The Netherlands

**Keywords:** Continuing medical education, CPOE, Information technology, Meaningful use, Medication management, Physicians

## Abstract

**Background:**

Using information technology for medication management is an opportunity to help physicians to improve the quality of their documentation and communication and ultimately to improve patient care and patient safety. Physician education is necessary to take full advantage of information technology systems. In this trial, we seek to determine the effectiveness of an intensive educational intervention compared with the standard approach in improving information technology–mediated medication management and in reducing potential adverse drug events in the outpatient clinic.

**Methods/Design:**

We are conducting a multicenter, cluster randomized controlled trial. The participants are specialists and residents working in the outpatient clinic of internal medicine, cardiology, pulmonology, geriatrics, gastroenterology and rheumatology. The intensive educational intervention is composed of a small-group session and e-learning. The primary outcome is discrepancies between registered medication (by physicians) and actually used medication (by patients). The key secondary outcomes are potential adverse events caused by missed drug–drug interactions. The primary and key secondary endpoints are being assessed shortly after the educational intervention is completed. Sample size will be calculated to ensure sufficient power. A sample size of 40 physicians per group and 20 patients per physician will ensure a power of >90 %, which means we will need a total of 80 physicians and 1,600 patients.

**Discussion:**

We performed an exploratory trial wherein we tested the recruitment process, e-learning, time schedule, and methods for data collection, data management and data analysis. Accordingly, we refined the processes and content: the recruitment strategy was intensified, extra measures were taken to facilitate smooth conductance of the e-learning and parts were made optional. First versions of the procedures for data collection were determined. Data entry and analysis was further standardized by using the G-standard database in the telephone questionnaire.

**Trial registration:**

ISRCTN registry: ISRCTN50890124. Registered 10 June 2013.

## Background

Treating patients with medication is one of the core activities of physicians in the outpatient clinics of internal medicine and related specialties. Unfortunately, adverse drug events (ADEs) frequently occur in this setting [1, 2]. Patients are often treated simultaneously for more than one medical condition, by more than one physician, with more than one drug and use various pharmacies to obtain prescribed and non-prescribed medications. This may result in scattering of information about the patient’s previous and actual medication use and allergies. One of the causes of ADEs is a discrepancy between what the physician thinks the patient is taking and what the patient is actually taking at home [3]. To prevent these discrepancies and resulting medical consequences, medication should be appropriately managed by physicians in the outpatient setting. This includes appropriate documentation of the medication, appropriate communication to other health care providers and engaging patients in management of their own medication [4, 5].

Using information technology (IT) for medication management is an opportunity to support physicians to improve the quality of their documentation and communication and ultimately to reduce ADEs. IT for medication management refers to the combination of computerized physician order entry (CPOE) and clinical decision support systems.

To stimulate the improvement of quality of care by use of IT, “meaningful use” criteria are used in the United States. With respect to CPOE, these criteria include maintaining an active medication and allergy list for each patient and providing all patients with a copy of their medication information [4]. In the Netherlands, a guideline, “handover of medication information between care settings”, has come into force [6]. This guideline advises physicians to be aware of which medications the patient is taking and to provide patients with a printed overview of their current medications, recent changes and the reasons therefor.

Education of users (that is, physicians) is necessary to improve medication management and to take full advantage of IT systems. In addition, inappropriate use of IT may lead to new kinds of errors [6].

The objective of the present study is to determine the effectiveness of an intensive educational intervention compared with the usual approach in improving IT-mediated medication management and in reducing ADEs in the outpatient clinic. This objective pertains to the cluster (physician) level.

## Methods/Design

The Standard Protocol Items: Recommendations for Interventional Trials (SPIRIT) 2013 guidelines were used to write the study protocol [7] We are conducting a two-arm cluster randomized superiority trial, with a 1:1 allocation ratio in two academic hospitals. Physicians are the unit of allocation. The intervention is targeted on the cluster (physician) level.

Physicians are randomly allocated either to the control group, who have already received the “usual approach”, or the intervention group, who also have already received the usual approach and now will receive an additional intensive educational intervention. Randomization will be done by using a computerized system with randomly permutated blocks. A small block size of six clusters per block will be chosen because the clusters are recruited and randomized sequentially. Randomization will be stratified by hospital to balance the influence of the geographic areas, work environments and IT systems of the two hospitals [8].

A cluster randomization design was adopted because the intervention is at the level of the physician and there is a risk of contamination at the level of the physician. Physicians trained in a new technique will find it difficult to revert to an old technique at the toss of a coin. Allowing physicians to deliver similar care to similar patients ensures that the trial is following typical clinical practice more closely [8].

### Setting

The study will be conducted in outpatient settings of internal medicine departments and related specialties and subspecialties at two academic hospitals in the Netherlands: University Medical Center Utrecht (UMCU) and Erasmus University Medical Center Rotterdam (EMC). The study is focused on internal medicine physicians because pharmacotherapy is their core treatment option.

CPOE systems are available in the two participating hospitals. UMCU uses the ChipSoft hospital information system (ChipSoft BV, Amsterdam, the Netherlands), in which the CPOE is fully integrated into the electronic health record. EMC uses a CPOE system called iSoft Medicator (Computer Sciences Corp (CSC), Groningen, the Netherlands), which is partly integrated into the electronic health record. Both CPOE systems meet the basic requirements of CPOE systems, which are ability to store a current medication list and allergies and basic decision support (drug–drug interaction, dose, duplicate order, contraindications) [9].

The ethical review boards of both academic hospitals reviewed the protocol and declared that this research does not fall under the Dutch legislation for research on human subjects, because of its limited burden on participating patients. The ethical review board of the Dutch organization of medical education approved the protocol and telephone questionnaire with respect to scientific content and accuracy.

### Participants: eligibility, recruitment, informed consent and time line

#### Inclusion criteria for physicians

- Specialists and residents of internal medicine (including general internal medicine, nephrology, endocrinology, infectious diseases, oncology, hematology, vascular medicine, inherited and metabolic diseases, and acute internal medicine), cardiology, pulmonology, geriatrics, gastroenterology and rheumatology
- Physicians with consultations in the outpatient clinic for at least 4 hours per week

#### Exclusion criterion for physicians

- Physicians who were involved in the development of the intensified educational intervention being investigated

#### Inclusion criteria for patients

- Patients older than 18 years of age
- Patients visiting the outpatient clinic consulting a physician who is participating in the study

#### Exclusion criteria for patients

- Patients who are unable to understand and speak Dutch or English
- Patients who have insufficient understanding of their medications to answer questions about their medicine, or patients who do not have a caregiver who can answer the questions

Physicians are recruited by giving a talk about the study in a meeting with eligible physicians. During the talk, physicians are given the information letter, and they are asked to participate in the study. They are asked to sign a consent form if they decide to participate. Physicians in the intervention and control groups will receive the same information letter and informed consent form. Physicians in our study are not fully informed about the measurements of the (patient-related) outcomes, because this information could influence the behavior of participants in the control group. This approach was subject to the approval provided by the ethical review board.

Consecutive patients who visit a participating physician for an outpatient consultation during the enrollment period are asked in a telephone questionnaire to give information about their medication use, and they are asked to give permission for access to their medical records.

There will be two ways of asking patients to participate: by a research assistant present in the waiting room or by medical assistants. To avoid physicians’ changing their behavior because they know patients are included, the inclusion process is as non-intrusive as possible.

When patients immediately agree to participate, they sign the informed consent right away. Patients who consider participation will be called in the week ahead. If they agree to participate, they are asked to return the informed consent by mail.

In our study, the physician represents the cluster. The intervention is targeted on the physician who cares for individual patients. This holds that patients are not able to avoid a treatment of a physician who received or did not receive the intervention. However, they are able to choose whether to participate in the telephone questionnaire and whether to give us permission for access to their medical record.

To ensure a representative sample of patients per physician, no more than five patients per day per physician will be asked to participate. To minimize the chance of interim changes in their medications, patients will be called as soon as possible, with a maximum lapse of 2 weeks after the index visit at the outpatient clinic.

**Fig. 1:**
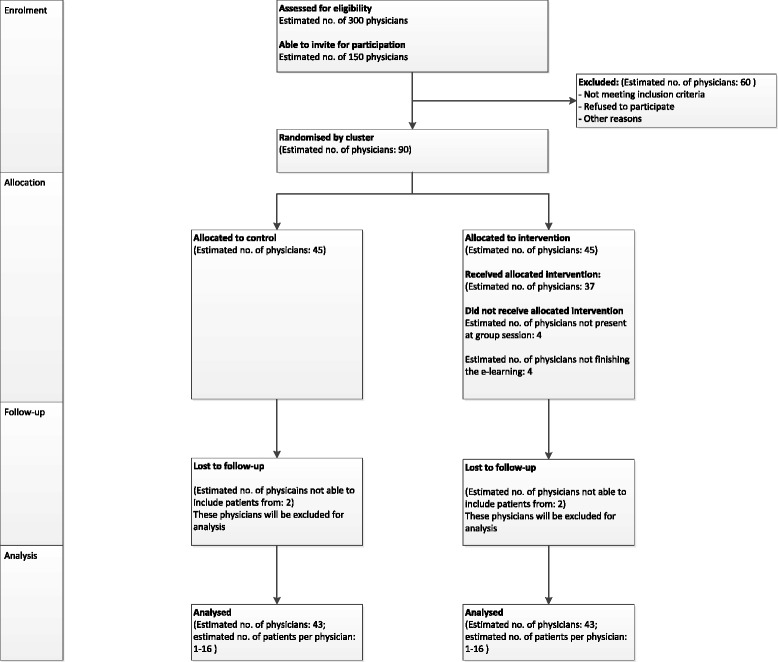
Patient flow through the trial

Participant flow is illustrated in Fig. 1. The measurements for the primary and key secondary outcomes will take place in groups of physicians to allow for flexible planning. After completion of the educational intervention, consecutive patients of these physicians will be invited to participate in the study. The enrollment period is also flexible because of the variations in numbers of patients a particular physician sees in the outpatient clinic.

Electronic assessment of knowledge and skills will be carried out in three groups to allow for approximately equal time between randomization and assessment. The intervention group will be assessed at a time point approximately 6 months after completing the educational intervention. The control group will be assessed at a time point approximately 6 months after the median number of months whereafter the intervention was completed after randomization by physicians in the intervention group, with the control assessment time point being the median number of months between randomization and completion of education by intervention group plus 6 months).

### Blinding

Physicians are randomly allocated right after inclusion by the investigator, following the assignment generated by the computer system. As the assignment for the next allocation is provided at the moment of randomization, allocation is sufficiently concealed. Of course, the intervention itself is not blinded to the participating physicians.

Patients are blinded to the intervention status of their physicians. Physicians are not fully informed about the nature of the patient-related outcome measures. Researchers who mediate the group sessions are aware of the intervention status of the physicians. Research assistants are aware of the intervention status of physicians, but they are not knowledgeable about the content of the educational intervention. The researcher who is analyzing the data does not collect the data. Data analysis will be undertaken blinded to study arm allocation; that is, the control and intervention will be identified only as “A” and “B” until analysis is complete.

### Intervention

#### Usual approach as control

In the context of this study, the usual approach is the instruction that is currently given when IT for medication management is made available to physicians. Usually, classroom instruction consisting of a lecture demonstrating the main features of the system is offered, and limited opportunities for practical exercises exist. In this study, the usual approach was not standardized between hospitals and physicians.

#### Usual approach plus intensive educational intervention

In addition to the usual instruction, physicians in the intervention arm will receive an intensive educational intervention consisting of a small-group session and e-learning.

Physicians will attend a small-group session with discussions on the advantages and disadvantages of IT-mediated medication management to establish mutual agreements between professionals about how the medication management task is properly performed. There will be also opportunities to share experiences, ask questions and discuss cases. There will be approximately eight physicians in the group session, which will last approximately 1 hour.

In the e-learning, physicians will be informed about the expected benefits and limits of IT to adequately calibrate their trust in IT and form an appropriate attitude toward IT. A social norm will be set for effective and safe IT-mediated management by role modeling of experts on video. The training is built up from easy to difficult cases to promote self-efficacy. Physicians are given step-by-step procedural instructions and information to support problem solving. The training will be tailored because it is self-directed. Physicians may choose their starting level, to practice a case again, to practice a specific self-chosen part of a task again or to go on to the next level of difficulty. This part of the intervention will be individually delivered as e-learning modules developed according to the four-component instructional design (4C/ID) model [10]. The 4C/ID model allows for research-based design of educational interventions. The e-learning is generic, but specific procedures in CPOE are tailored to the actual local CPOE system in use [11].

To stimulate adherence to the intensive educational intervention, physicians in the intervention group receive several reminders. Earning continuing medical education credits when both the small-group session and the e-learning modules are finished further stimulates adherence.

### Outcomes

#### Primary outcome: medication discrepancies

Differences between the two study arms are analyzed using as the primary outcome the proportion of medication discrepancies per physician. *Medication discrepancies* are defined as discrepancies between medications registered by physicians in CPOE and medications actually used by patients, obtained by the telephone questionnaire. Each discrepancy is counted; that is, any patient can contribute multiple discrepancies.

The following events are taken into account:

- *Omission in registration of the current medication*: A drug is taken by the patient but is not registered in the list of medications in CPOE
- *Addition in the registration of the current medication*: A drug is not used by the patient but is registered in CPOE.

Discrepancies in dosing (dose per intake and number of intakes per unit of time) are not taken into account.

The proportion of discrepancies is then calculated using (1) the number of discrepancies (omissions and additions) as the numerator and (2) the sum of the number of discrepancies and the number of medications a patient is taking and the number of medications registered in CPOE minus the overlap as the denominator. Thus, no medication is counted twice in the denominator.

The proportion of discrepancies will also be determined and analyzed, restricted to high-risk medications specifically. The high-alert medication list of the Institute for Safe Medication Practices will be used to indicate high-risk medications [12].

The data collected by means of the telephone questionnaire are considered the gold standard for the patient’s use of medication and past ADEs. The telephone questionnaire is derived from the structured medication history [5], the telephone questionnaire used by Gandhi *et al*. [1] and Consumer Quality Index questions tailored for individual patient experiences in health care [13]. A prior version of the telephone questionnaire was validated against the medication history obtained during home visits. Improvements were made accordingly.

Data collectors are medical students or medical doctors in the phase just before or after their graduation or should be experienced research nurses. Data collectors are trained in two ways. First, there is a standard operating procedure (SOP) describing how exactly the questionnaire should be carried out and what to do in various circumstances (for example, when a caregiver answers the questions). Second, data collectors work their first day together with an experienced data collector and have opportunities to practice and receive feedback.

The telephone questionnaire will be made available at the trial website only after completion of data collection to ensure blinding of participating physicians for the outcome measures involved [14]. Data managers extract the following data from the CPOE systems: medication on the index date and registered ADEs.

#### Key secondary outcome: missed drug–drug interactions with potential for causing harm

The key secondary outcome is any difference between the two study arms in the proportion of patients per physician with at least one missed drug–drug (DD) interaction with potential for causing an ADE (PADEs) per physician. Whether there is a DD interaction with potential for an ADE is assessed by following the Dutch clinical guidelines for management of DD interactions using the G-standard database. The G-standard is the Dutch drug database, which is used by all Dutch parties in health care, including physicians, pharmacists, manufacturers, health insurers and the government. The G-standard supports the different processes in health care, such as, among others, decision support on DD interactions. Also, information is given about the (published) effect of the interaction. The G-standard categorizes the effects of the interactions into six levels of severity ranging from A (minor) to F (potentially lethal) [15].

#### Other secondary outcome measures

##### Discrepancies in adverse drug events

Differences between the two study arms in the proportion of patients with at least one missed ADEs per physician are defined as discrepancies between the registration of ADEs in CPOE versus the information regarding ADEs obtained from the patient via the telephone questionnaire. We take into account the ADEs with moderate or severe potential consequences. The European Medicines Agency guideline will be used to assess the severity of the ADE [16].

##### Use of computerized physician order entry

Physicians’ use of CPOE will be registered with log files of the CPOE systems to assess whether physicians meet the official meaningful use criteria [4]. Also, additional criteria were formulated to assess meaningful use. The following aspects will be assessed:

- The number of lines inserted in CPOE per week (numerator) divided by the number of patients scheduled for outpatient consultation per week (denominator)
- The number of allergies inserted in CPOE per week (numerator) divided by the number of patients scheduled for outpatient consultation per week (denominator)
- The number of complete ADE registrations (medication, symptoms, severity) inserted in CPOE per week (numerator) divided by the number of ADEs inserted in CPOE per week (denominator)
- The number of prescriptions with unspecified doses in CPOE (numerator) divided by the number of lines inserted in CPOE per week (denominator) (In the numerator, we will exclude medication obliged to be prescribed this way, because doses are adjusted to frequent measurements, such as insulin and vitamin K antagonists.)

CPOE use will be logged 2 months before the intervention, during the intervention and until 6 months after the intervention. These data will give an indication whether the intervention remains effective over a longer period of time (that is, retention of learning).

Data derived from the telephone questionnaire supports the analysis of the physicians’ adherence to the Dutch guidelines regarding handover of medication information between care settings by calculating the difference between study arms in the following terms:

- Proportion of patients who were actively asked for their use of medication during the consultation
- Proportion of patients who were given a printed medication overview at the end of the consultation

#### Knowledge and skills

With an assessment after the intervention period, physicians’ knowledge and skills regarding IT-mediated medication management will be measured. To ensure content validity of the assessment, a test matrix is used to guarantee an even distribution of training content in the questions. The checklist for constructing written test questions for basic and clinical sciences will be used to ensure high-quality questions [17]. An expert team of an educator, pharmacist, clinical pharmacologist and internist will be asked to review the questions and propose improvements. The distinctiveness of the assessment will be determined by asking an expert and a naive end user to pretest the assessment. Improvements will be made according to the findings.

#### Patient-related outcomes

Patient-related outcomes will be analyzed on the basis of the data derived from the telephone questionnaire by calculating the difference between study arms in terms of

- Patients’ satisfaction with their care regarding their medications
- Patients’ sense of responsibility to self-manage their medications

### Sample size calculation

For the sample size considerations, the following assumptions are made. Per specialist and per period, the same number of patients are followed. The primary outcome is the percentage of discrepancies between medication records and patient information. The design is a cluster (physician) randomized trial with proportions as outcomes. The comparison between groups should be able to detect a difference of at least 10 % (from 70 % to 80 % and from 80 % to 90 %), and testing is done at a significance level of 5 % (two-sided) for each contrast. The intraclass correlation coefficient is assumed to be 0.1, following the method of Schnipper *et al*. [18]. To ensure sufficient power, a sample per group of 40 physicians and 20 patients will ensure a power of >90 % for a total of 80 physicians and 1,600 patients. If (substantially) more physicians can be recruited, this may lead us to recruit fewer patients while maintaining power.

### Statistical methods

#### Descriptive statistics

We will use baseline characteristics of hospitals, physicians and patients according to covariates known from the literature or anticipated covariates, including, among others, physician’s age, sex, years of experience in prescribing and computer literacy, as well as patient’s number of medications [18–21].

#### Comparisons between study arms

The primary analyses will be focused on comparing the two study arms with regard to the proportions of discrepancies and resulting PADEs. The unit of analysis is the cluster (physicians). The analysis will follow the intent-to-treat principle, including all physicians in the analysis as randomized. However, physicians who do not contribute any patients (and hence contribute no observations) will be excluded from the analysis. No imputation of resulting missing values will be done.

The primary outcome per patient is the proportion of discrepancies, as defined above. A full regression model appropriate to the structure of the data could be applied. However, the proportions as defined most likely cannot be modeled as binomial quantities, but a continuous approximation may be sufficiently adequate if the average proportion per cluster (physician) is analyzed. This analysis is somewhat less efficient than a full model (if that can be correctly specified), but cluster effects are appropriately included in the error term. Hence, this linear model will be used to analyze the data (potentially appropriately transformed if model evaluation so indicates).

The population-averaged model with inclusion of the covariate can be written algebraically as follows:

$$ {\mathit{\mathsf{Y}}}_{\mathit{\mathsf{i}}\mathit{\mathsf{j}}\mathit{\mathsf{h}}}=\mathit{\mathsf{\alpha}}+\mathit{\mathsf{\beta}}{\mathit{\mathsf{x}}}_{\mathit{\mathsf{i}}}+{\mathit{\mathsf{z}}}_{\mathit{\mathsf{h}}}+{e}_{\mathit{\mathsf{i}}\mathit{\mathsf{j}}\mathit{\mathsf{h}}}, $$

where *i* is the intervention arm, *j* is the cluster (physicians), *h* is the Hospital, *Y*_ijh_ indicates the value of the average outcome for the *i*th treatment arm from the *j*th physician of hospital *h*, α is the constant, β is the effect size of the intervention, *x*_i_ indicates the intervention arm (value 1 = intervention arm, value 0 = control arm), *z*_h_ indicates the categorical covariate (*h*th hospital), and *e*_ijh_ is the residual for *j*th physician in treatment arm *i* and hospital *h*.

The analysis of secondary endpoints is exploratory and will follow the same analytical approach used for the primary endpoint. Because of the exploratory nature of the analysis, no type I error correction for multiplicity will be done.

#### Data management

The data are collected by telephone and entered at the core coordinating center (UMCU) in Research Online 2 (RO2). RO2 is an electronic data capture system that will be used for data collection. Integrity is enforced when data entry takes place in the web-based case report forms by means of checks on value ranges, logical checks (for example, the interview date cannot be later than the actual date of interview), skip rules preventing unnecessary data collection and coded answering options. To ensure analogy between data entry in RO2 and CPOE, the G-standard database is implemented in RO2.

RO2 data traffic over the Internet is encrypted using secured data communication protocols. Dedicated databases and web servers are hosted in data centers that meet the highest available standards for security (XS4ALL data center, Diemen, the Netherlands: https://www.xs4all.nl/).

Access to and exporting of the RO2 data are limited to data management staff of the Julius Center of UMCU, who are not involved in the conduct of the trial. The patients’ data are coded, and the linking codes are stored in separate password-protected locations. The data will be prepared for analysis and stored on a secure server within the IT facilities at UMCU. Access to the specific folders on this server containing the research data is kept to a minimum and granted only to researchers who are specified by the principal investigator. Data will be locked at the end of the study and securely stored for 15 years. Only after the principal investigator has granted permission the data will be allowed to be unlocked.

Files with patient-related data will be sent from one academic center to the core coordinating center. These files will be sent using FileSender (SURFnet: https://filesender.surfnet.nl/), a specially secured way to send data with passwords. Files are zipped and encrypted before sending.

## Discussion

We performed an exploratory trial wherein we tested the following:

1. Recruitment process
2. Technical smoothness of the e-learning
3. Physicians’ reactions to the e-learning
4. Potential room for improvement regarding the primary outcome
5. Appropriateness of the time schedule
6. Data collection methods
7. Data management
8. Categorization of medication and interaction discrepancies

We refined the processes and content according to our findings as follows:

1. We intensified the recruitment strategy.
2. We gave special warnings for certain systems hindering the smoothness of e-learning and introduced the possibility of carrying out the first module together with the research physician.
3. Physicians were mostly positive, but they found the e-learning too long. Therefore, we made some of the content of the e-learning optional.
4. It turned out that there was enough potential room for improvement, because only 37 % of the medication records in the exploratory trial were complete. After finishing the e-learning, most physicians reported their intent to change their behavior regarding IT-mediated medication management or had already changed their behavior.
5. Physicians needed more time to finish the e-learning.
6. First versions of the SOPs for data collection were determined.
7. Data entry was further standardized by using the G-standard database in the telephone questionnaire.
8. Improvements in categorization were made, mostly with the automation of the categorization process.

## Trial status

Trial recruitment was completed on 1 January 2015.

## Abbrevations

ADE: Adverse drug event
4C/ID: Four-component instructional design
CPOE: Computerized physician order entry
DD: Drug–drug; EMC
Erasmus Medical Center: Rotterdam, the Netherlands
IT: Information technology
MRC: Medical Research Council
PADE: Potential adverse drug event
RO2: Research Online 2
SOP: Standard operating procedure
UMCU: University Medical Center Utrecht, Utrecht, the Netherlands

## References

[1] Gandhi TK, Weingart SN, Borus J, Seger AC, Peterson J, Burdick E (2003). Adverse drug events in ambulatory care. N Engl J Med.

[2] Avery T, Barber N, Ghaleb M, Franklin BD, Armstrong S, Crowe S, et al. Investigating the prevalence and causes of prescribing errors in general practice: the PRACtiCe Study (PRevalence And Causes of prescribing errors in general practiCe): a report for the GMC. London; General Medical Council; May 2012. http://www.gmc-uk.org/Investigating_the_prevalence_and_causes_of_prescribing_errors_in_general_practice___The_PRACtICe_study_Reoprt_May_2012_48605085.pdf. Accessed 15 May 2015.

[3] Kohn LT, Corrigan JM, Donaldson MS, editors; Committee on Quality of Health Care in America, Institute of Medicine. To err is human: building a safer health system. Washington, DC, National Academy Press; 2000. https://www.iom.edu/~/media/Files/Report%20Files/1999/To-Err-is-Human/To%20Err%20is%20Human%201999%20%20report%20brief.pdf. Accessed 15 May 2015.

[4] Blumenthal D, Tavenner M (2010). The “meaningful use” regulation for electronic health records. N Engl J Med.

[5] Drenth-van Maanen AC, Spee J, van Hensbergen L, Jansen PA, Egberts TC, van Marum RJ (2011). Structured history taking of medication use reveals iatrogenic harm due to discrepancies in medication histories in hospital and pharmacy records. J Am Geriatr Soc.

[6] Koppel R, Metlay JP, Cohen A, Abaluck B, Localio AR, Kimmel SE (2005). Role of computerized physician order entry systems in facilitating medication errors. JAMA.

[7] Chan AW, Tetzlaff JM, Altman DG, Laupacis A, Gøtzsche PC, Krleža-Jerić K (2013). SPIRIT 2013 statement: defining standard protocol items for clinical trials. Ann Intern Med.

[8] Eldridge S, Kerry S (2012). A practical guide to cluster randomised trials in health services research.

[9] van der Sijs H, Bouamar R, van Gelder T, Aarts J, Berg M, Vulto A (2010). Functionality test for drug safety alerting in computerized physician order entry systems. Int J Med Inform.

[10] van Merriënboer JJG, Kirschner PA (2013). Ten steps to complex learning, a systematic approach to four-component instructional design.

[11] Grol R, Wensing M (2006). Implementatie: effectieve verbetering van de patiëntenzorg.

[12] Institute for Safe Medication Practices (ISMP). ISMP list of high-alert medications in community/ambulatory care. http://www.ismp.org/communityRx/tools/highAlert-community.pdf. Accessed 15 May 2015.

[13] BMJ Outcomes. Consumer Quality Index – measuring patient experience in the Netherlands. http://outcomes.bmj.com/index.php/journal/consumer-quality-index-measuring-patient-experience-in-the-netherlands. Accessed 15 May 2015.

[14] UMC Utrecht. MEDUCATE trial. http://www.umcutrecht.nl/en/Ziekenhuis/Meedoen-aan-wetenschappelijk-onderzoek/MEDUCATE-trial. Accessed 15 May 2015.

[15] van Roon EN, Flikweert S, le Comte M, Langendijk PN, Kwee-Zuiderwijk WJ, Smits P (2005). Clinical relevance of drug–drug interactions: a structured assessment procedure. Drug Saf.

[16] European Medicines Agency. http://www.ema.europa.eu/docs/en_GB/document_library/Scientific_guideline/2009/09/WC500002749.pdf. Accessed 15 May 2015.

[17] National Board of Medical Examiners. http://www.nbme.org/. Accessed 15 May 2015.

[18] Schnipper JL, Hamann C, Ndumele CD, Liang CL, Carty MG, Karson AS (2009). Effect of an electronic medication reconciliation application and process redesign on potential adverse drug events: a cluster-randomized trial. Arch Intern Med.

[19] Harrison MI, Koppel R, Bar-Lev S (2007). Unintended consequences of information technologies in health care–an interactive sociotechnical analysis. J Am Med Inform Assoc.

[20] Linder JA, Rigotti NA, Schneider LI, Kelley JH, Brawarsky P, Schnipper JL (2011). Clinician characteristics and use of novel electronic health record functionality in primary care. J Am Med Inform Assoc.

[21] Leendertse AJ, Egberts AC, Stoker LJ, van den Bemt PM, HARM Study Group (2008). Frequency of and risk factors for preventable medication-related hospital admissions in the Netherlands. Arch Intern Med.

